# Image Restoration for Fluorescence Planar Imaging with Diffusion Model

**DOI:** 10.1155/2017/2010512

**Published:** 2017-11-27

**Authors:** Xuanxuan Zhang, Yuzhu Gong, Yang Li, Xu Cao, Shouping Zhu

**Affiliations:** Engineering Research Center of Molecular and Neuro Imaging of the Ministry of Education & School of Life Science and Technology, Xidian University, Xi'an, Shaanxi 710071, China

## Abstract

Fluorescence planar imaging (FPI) is failure to capture high resolution images of deep fluorochromes due to photon diffusion. This paper presents an image restoration method to deal with this kind of blurring. The scheme of this method is conceived based on a reconstruction method in fluorescence molecular tomography (FMT) with diffusion model. A new unknown parameter is defined through introducing the first mean value theorem for definite integrals. System matrix converting this unknown parameter to the blurry image is constructed with the elements of depth conversion matrices related to a chosen plane named focal plane. Results of phantom and mouse experiments show that the proposed method is capable of reducing the blurring of FPI image caused by photon diffusion when the depth of focal plane is chosen within a proper interval around the true depth of fluorochrome. This method will be helpful to the estimation of the size of deep fluorochrome.

## 1. Introduction

Fluorescence imaging techniques have become indispensable tools for numerous biomedical applications attributing to the everlasting development of fluorescent probes [1]. With the help of various fluorescent probes and fluorescence reporter techniques [2, 3], fluorescence imaging techniques are capable of tracing biomedical processes at cellular and subcellular levels* in vivo* and noninvasively in wide applications such as gene expression, protein function, and cell therapy [4–8].

Up to the present, a number of fluorescence imaging techniques have been developed [9–15]. Microscopic fluorescence imaging techniques provide high spatial resolutions but suffer from small fields of vision. On the contrary, macroscopic fluorescence imaging techniques can capture whole-body images for small animals but with a limited spatial resolution. Fluorescence planar imaging (FPI) [16–20] is the most widely used macroscopic fluorescence imaging technique, which directly detects the fluorescence photons on the surface of an imaged small animal using camera. According to the locations of excitation light source and camera, FPI can be formed in two different modes [1, 21]: epi-illumination mode and transillumination mode. Epi-illumination mode places excitation source and camera at the same side of the imaged small animal, which collects fluorescent photons in the same direction of the reflected excitation lights; thus it is also called fluorescence reflectance imaging (FRI). The defect of this mode is the difficulty of the excitation of deep fluorochromes. As an alternative, transillumination mode places the imaged small animal between excitation light source and camera. This mode can easily excite the fluorochromes far away from camera but the images are more heavily contaminated by excitation lights than epi-illumination mode although the excitation lights are attenuated by filters.

Whichever mode is applied, FPI is incapable of imaging deep fluorochromes with high spatial resolution. It is well-known that the penetration depth of near-infrared light in tissues is several centimeters [22]. Nevertheless, due to the elastic scattering, near-infrared photons are diffused after several millimeters of propagation in tissues [23]. So the fluorescent images acquired with camera are blurred. The deeper the fluorochromes are, the more strongly the fluorescent photons are diffused and the more blurry the images are. This restricts the applications of FPI in many cases. For instance, when imaging deep tumors, it is difficult to estimate the sizes of tumors through FPI images because they are strongly blurred.

Image restoration techniques aim to eliminate or reduce the impact of image degradation such as blurring. The causations of blurring can be classified into three types [24]: medium-induced, optical, and mechanical. The blurring derived from photon diffusion belongs to the first and second types due to the elastic scattering in medium. Blurring can be described with linear or nonlinear models, which depends on the specific problem. The general linear model can be summarized as *u*_0_ = *Ku* + *n* [24–26], where *n* denotes noise, *K* is the system matrix, and *u*_0_ and *u* are the blurry and expected images, respectively. The key of deblurring is the construction of *K*, which is known as the point spread function (PSF) in many applications. Because the linear model is usually formed with a convolution like *Ku* = *k∗u*(*x*) [24–26], deblurring is also called deconvolution. During the last two decades, the researches of deblurring in fluorescence imaging focused on microscopic fluorescence imaging techniques [26–33] which are known as techniques with almost no photon diffusion. In these investigations, researchers implemented deconvolution methods to deal with the blurring derived from imaging system, that is, the mechanical type of blurring through PSFs of imaging system.

In this paper, we aim to build a method to reduce the impact of the blurring derived from photon diffusion in FPI. This will be helpful to the estimation of the size of deep fluorochrome. The scheme of the proposed image restoration method is conceived based on a reconstruction scheme in fluorescence molecular tomography (FMT) [34–37], in which the diffusion model [38–40] is used to describe the photon propagation in tissues, the Born approximation [41–43] is applied to solve the diffusion equation, and the Kirchhoff approximation [44, 45] is implemented to obtain Green's function. Different from the blurring in fluorescence microscopic imaging, the blurring in FPI is not caused by imaging system. Consequently, the construction method of system matrix in fluorescence microscopic imaging is not applicable to the deblurring in FPI. The primary contribution of this work is the construction of the system matrix for FPI. Through introducing the first mean value theorem for definite integrals, we define a new unknown parameter as the restoration target rather than the fluorescent yield. The new unknown parameter is a weighted average of the voxel values of fluorescent yield along detection direction. To construct the system matrix that converts this parameter to the blurry image, depth conversion matrix is defined, which consists of the weights of the voxels with different depths related to the same pixel of the expected image. Subsequently, the elements of depth conversion matrices related to a chosen plane named focal plane are selected to construct the system matrix according to a proportional relationship. Finally, the Levenberg-Marquardt method [46, 47] is applied to solve the system equation and acquire the restored image. Phantom and mouse experiments are carried out to validate the proposed method.

## 2. Methods

The generation of fluorescence consists of two processes: excitation and emission. In the excitation process, photons from excitation source propagate to fluorochromes. Subsequently, fluorescent photons emitted from fluorochromes propagate to detectors in the emission process. Each process can be modeled by the diffusion equation with Robin-type boundary condition as follows [35–39]:

(1) −∇·Dr∇Φr+μarΦr=Qrr∈Ω2qDr∂Φr∂n→+Φr=0r∈∂Ω,

where *r* denotes the position, Φ is the photon density, *Q* is the source term, and *μ*_*a*_ and *D* are the absorption and diffusion coefficients, respectively. The diffusion coefficient *D* is defined as *D* = 1/[3(*μ*_*a*_ + *μ*_*s*_′)], where *μ*_*s*_′ is the reduced scattering coefficient. Ω is the domain of the object and *∂*Ω is the corresponding boundary. $\vec{n}$ denotes the outward normal vector and *q* is a coefficient related to the reflective index mismatch at boundary [40]. For the excitation process, the source term *Q* is determined by the location of excitation source and commonly approximated as an isotropy point source located one scattering length below the surface when a collimated source is used [39, 40]. As for the emission process, the source term *Q* is determined by the distribution of the photon density for excitation as well as the fluorescent yield of fluorochrome.

In this paper, the Born approximation [41–43] is used to solve the diffusion equations as follows:

(2) $$\begin{array}{c} \Phi \left(\;r_{d},r_{s}\;\right)=\overset{}{\underset{\Omega }{\int }}G_{em}\left(\;r_{d},r\;\right)f\left(\;r\;\right)\Phi_{ex}\left(\;r,r_{s}\;\right)dr, \end{array}$$

where *G* is Green's function solution to the diffusion equation and *f* is the fluorescent yield of fluorochrome. *G*_em_(*r*_*d*_, *r*) denotes the photon density for emission at the position of detector *r*_*d*_ when a point source is located at position *r*. Φ_ex_(*r*, *r*_*s*_) denotes the photon density for excitation at position *r* when the source is located at *r*_*s*_. If the excitation source is a point source, Φ_ex_(*r*, *r*_*s*_) is also a Green's function solution to the diffusion equation; otherwise, Φ_ex_(*r*, *r*_*s*_) is the convolution of Green's function and the distribution function of the source. Φ(*r*_*d*_, *r*_*s*_) is the fluorescent photon density for a pair of source and detector. The analytic formula of Green's function solution to the diffusion equation can be achieved only for infinite space, semi-infinite space, and several simple geometries. To obtain Green's function in geometries with arbitrary boundaries, the Kirchhoff approximation is implemented [44, 45].

**Figure 1:**
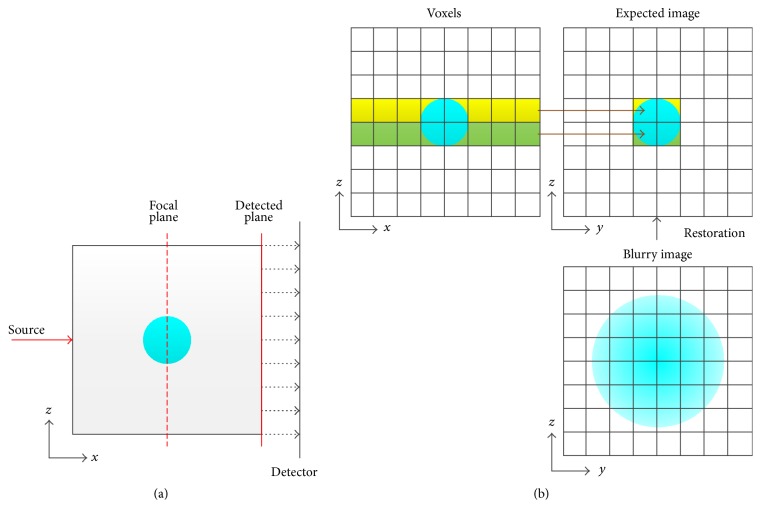
Schematic of the transillumination FPI and illustration of the image restoration problem. (a) Schematic of the transillumination FPI with a collimated source and planar detector. A sphere embedded in a cube is assumed to be the imaged fluorescent target. (b) Illustration of the image restoration problem.

Let us consider an imaging situation with transillumination mode as shown in Figure 1(a). A collimated source is used to excite fluorochrome and a planar detector is applied to capture fluorescent images. The imaged object is assumed to be a cube with a spherical fluorescent target located at the center. An illustration of the image restoration problem is shown in Figure 1(b). The fluorescent image acquired with the detector should be a blurry image due to the photon diffusion. The image we expect to achieve through the image restoration (hereinafter abbreviated as expected image) should be a projection along the detection direction, that is, *x*-axis.

Equation (2) can be written in a three-dimensional Cartesian coordinate system as follows:

(3) Φrd,rs=∬yzdy dz·∫xfx,y,zGemrd,x,y,zΦexx,y,z,rsdx.

Then we introduce the first mean value theorem for definite integrals:

(4) $$\begin{array}{c} \overset{b}{\underset{a}{\int }}f\left(\;x\;\right)g\left(\;x\;\right)dx=f\left(\;\varepsilon \;\right)\overset{b}{\underset{a}{\int }}g\left(\;x\;\right)dx, \end{array}$$

where *f*(*x*) is a continuous function on [*a*, *b*], *g*(*x*) is an integrable function that does not change sign on [*a*, *b*], and *ε* is a value in (*a*, *b*). Through (3) and (4), the following equation can be formed:

(5) Φrd,rs=∬yzfε,y,zdy dz·∫xGemrd,x,y,zΦexx,y,z,rsdx.

In (5), a new parameter *f*(*ε*, *y*, *z*) independent of *x*-axis displaces the fluorescent yield *f*(*x*, *y*, *z*). Subsequently, we discretize (5) with step lengths of Δ*x*, Δ*y*, and Δ*z* and obtain the following equation:

(6) Bm=ΔV∑n=1NPnm=ΔV∑n=1NCnmFn,Cnm=∑i=1NxCn,im=∑i=1NxGemrdm,xi,yn,znΦexxi,yn,zn,rs,Fn=fε,yn,zn,

where *B*_*m*_ = Φ(*r*_*dm*_, *r*_*s*_) is the value of the *m*th pixel of the blurry image, *r*_*dm*_ denotes the corresponding pixel location, *P*_*n*_^(*m*)^ is a component of *B*_*m*_ corresponding to the *n*th pixel of the expected image *F*_*n*_, Δ*V* = Δ*x* × Δ*y* × Δ*z* is the volume of voxel, and *N* = *N*_*y*_ × *N*_*z*_ is the number of pixels. *N*_*x*_, *N*_*y*_, and *N*_*z*_ are the numbers of voxels along *x*-axis, *y*-axis, and *z*-axis, respectively. *C*_*n*_^(*m*)^ is a weight that converts the expected image to the blurry image and *C*_*n*,*i*_^(*m*)^ is a component of *C*_*n*_^(*m*)^ after the discretization of *x*-axis where *i* is the index of *x*-coordinate.

**Figure 2:**
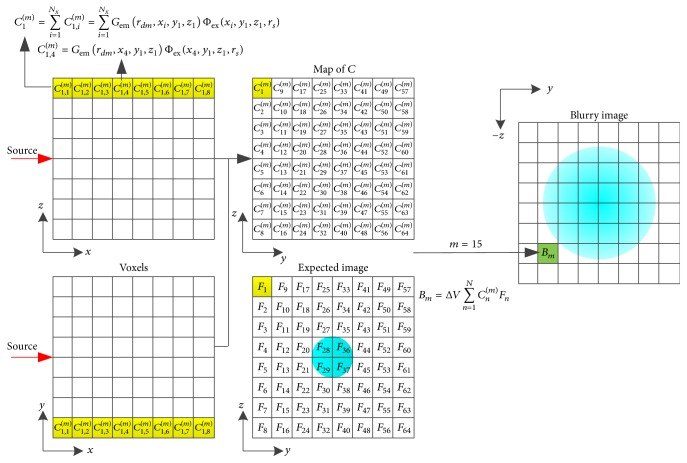
Illustration of the linear model described by (6) with an 8 × 8 image. The voxels in yellow correspond to the yellow elements in the map of *C* and the expected image. The map of *C* and the expected image relate to a pixel of the blurry image assumed to be the 15th pixel and colored in green.

An illustration of the linear model in (6) is shown in Figure 2, in which the image size is 8 × 8 and the pixel index of the blurry image *m* is assumed to be 15. Equation (6) describes the relationship between the expected image and the blurry image. The pixel values of the expected image *F*_*n*_ are weighted averages of the voxel values of fluorescent yield along *x*-axis, which can be described with

(7) $$\begin{array}{c} F_{n}=\frac{\sum_{i=1}^{N_{x}}C_{n,i}^{\left(m\right)}f\left(x_{i},y_{n},z_{n}\right)}{\sum_{i=1}^{N_{x}}C_{n,i}^{\left(m\right)}}. \end{array}$$

**Figure 3:**
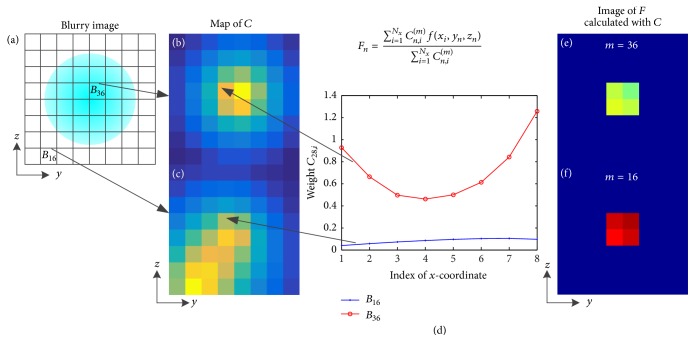
Illustration of the nonuniqueness of the expected image *F*. (a) An 8 × 8 blurry image. (b) and (c) Maps of weights *C* related to two different pixels (*B*_36_ and *B*_16_) of the blurry image. (d) Weights *C*_28,*i*_ for *B*_16_ and *B*_36_ as a function of index of *x*-coordinate. (e) and (f) Images of *F* calculated with *C* related to (b) and (c).

According to (6), the weight *C*_*n*,*i*_^(*m*)^ varies with the pixel location of the blurry image *r*_*dm*_. Consequently, based on (7) the pixel value of the expected image *F*_*n*_ is not the same for different pixels of the blurry image; that is, the expected image *F* is not unique.  Figure 3 illustrates the nonuniqueness of *F*. Figures 3(b) and 3(c) show the maps of *C* for two different pixels (*B*_36_ and *B*_16_) of the blurry image and Figure 3(d) gives profiles of *C*_28,*i*_ along *x*-axis for the two pixels. These figures indicate the differences between the weights corresponding to different pixels of the blurry image. Figures 3(e) and 3(f) are the images of *F* calculated with the weights *C* assuming that the fluorescent yield *f*(*x*_*i*_, *y*_*n*_, *z*_*n*_) is known, which show that the images of *F* are distinctly different for different pixels of the blurry image. In addition, the computational error results in the minute differences between the center four pixels. As a result of the nonuniqueness of *F*, it is infeasible to construct a system equation that converts the expected image *F* to the blurry image *B* through a combination of (6) for all the pixels of the blurry image.

In order to construct the system equation, firstly, we express (7) for all the pixels of the blurry image with the following matrix equation:

(8) Cn,11⋯Cn,Nx1⋮⋱⋮Cn,1N⋯Cn,NxNfx1,yn,zn⋮fxNx,yn,zn=Pn1⋮PnN=C11Fn1⋮CnNFnN.

Because *F* is not unique, we use a superscript on *F*_*n*_ to denote the differences caused by different pixels of the blurry image in (8). The matrix on the left of (8) consists of the weights corresponding to all the pixels of the blurry image and a certain pixel of the expected image. The elements of each row of this matrix are arranged according to the *x*-coordinate, that is, the depth. Each element determines the contribution of the fluorochrome at a certain depth to a certain pixel of the blurry image. Thus, we name this matrix depth conversion matrix.

To construct the system equation, we must build a set of equations that describe the relationship between the pixels of the blurry image and a stationary expected image. From (6), we know that the pixel values of the blurry image *B*_*m*_ are the summations of *P*_*n*_^(*m*)^. If we can displace the *F*_*n*_^(*m*)^ in (8) with the same *F*_*n*_, the relationship between the components *P*_*n*_^(*m*)^ and the pixel value of a stationary expected image *F*_*n*_ will be formed; then the system equation can be constructed. We achieve this purpose through a proportional relationship derived from (8) as follows:

(9) $$\begin{array}{c} P_{n}^{\left(j\right)}=C_{n}^{\left(j\right)}\frac{F_{n}^{\left(j\right)}}{F_{n}^{\left(1\right)}}F_{n}^{\left(1\right)}=C_{n}^{\left(1\right)}\frac{\sum_{i=1}^{N_{x}}C_{n,i}^{\left(j\right)}f\left(x_{i},y_{n},z_{n}\right)}{\sum_{i=1}^{N_{x}}C_{n,i}^{\left(1\right)}f\left(x_{i},y_{n},z_{n}\right)}F_{n}^{\left(1\right)}. \end{array}$$

In (9), the pixel value of the expected image is fixed as *F*_*n*_^(1)^ but the weight is changed. The fluorescent yield *f*(*x*_*i*_, *y*_*n*_, *z*_*n*_) is not known in image restoration. To obtain the weights, we manually choose a depth to approximate them as follows:

(10) $$\begin{array}{c} P_{n}^{\left(j\right)}=C_{n}^{\left(1\right)}\frac{C_{n,k}^{\left(j\right)}}{C_{n,k}^{\left(1\right)}}F_{n}^{\left(1\right)}, \end{array}$$

where the subscript *k* denotes the index of *x*-coordinate according to the chosen depth. The fluorescent signals are conceived to come from the plane at the chosen depth which is named focal plane.

**Figure 4:**
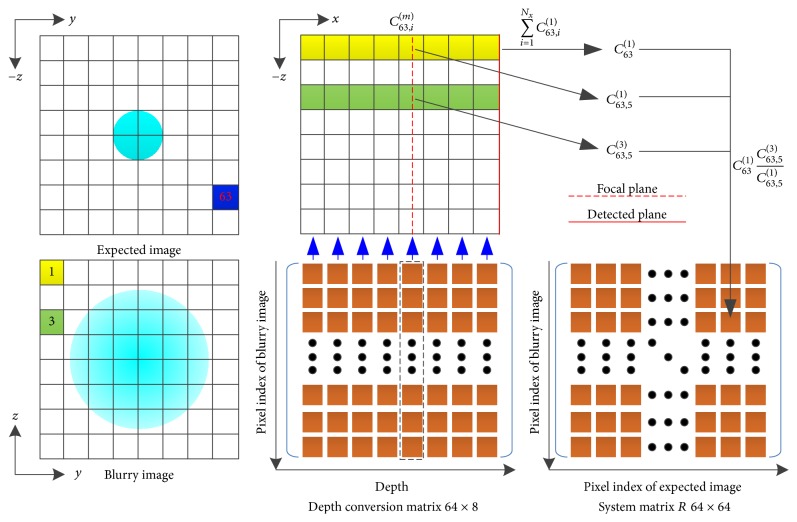
Illustration of the construction of system matrix. The calculation of the element of system matrix in the 3rd row and 63rd column is illustrated. Squares in blue and green denote the corresponding locations of the pixels of the expected image and the blurry image, respectively. The focal plane is shown with a red dotted line.

Based on (6) and (10), we can construct the system equation as follows:

(11) R11⋯R1N⋮⋱⋮RN1⋯RNNF11⋮FN1=B1⋮BN,

(12) Rmn=Cn1Cn,kmCn,k1,

where *R* is the system matrix that converts the expected image *F* to the blurry image *B*.  Figure 4 is an illustration of the construction of system matrix, which shows the calculation process of the element of system matrix in the 3rd row and 63rd column. Firstly, the depth conversion matrix of the 63rd pixel of the expected image is calculated through (6); then the elements at the 5th column are selected according to a chosen depth shown as the red dotted line in the top center subfigure and the elements of the 1st row are summed to calculate *C*_63_^(1)^; finally, the elements *R*_3,63_ of the system matrix are composed through (12). Although the system matrix is a square matrix, the inversion of *R* is an ill-posed problem in practical application. The Levenberg-Marquardt method [46, 47] is implemented to solve (11) as follows:

(13) $$\begin{array}{c} {\left.\;F\;\right|}_{n+1}={\left.\;F\;\right|}_{n}+{\left(\;R^{T}R+\lambda \alpha I\;\right)}^{-1}R^{T}\left(\;{\left.\;B-RF\;\right|}_{n}\;\right), \end{array}$$

where *F*|_*n*_ denotes the vector of the expected image for the *n*th iteration, *B* is the vector of the blurry image, *λ* is the regularization parameter, *α* is the trace of *R*^*T*^*R*, and *I* is the identity matrix.

In general, the image restoration consists of three steps. Firstly, the depth conversion matrices for all the pixels of the expected image are calculated. Subsequently, the system matrix is assembled with the depth conversion matrices and a chosen focal plane. Finally, the system equation is solved with the Levenberg-Marquardt method to achieve the restored image.

## 3. Experimental Setup

### 3.1. Phantom Experiments

**Figure 5:**
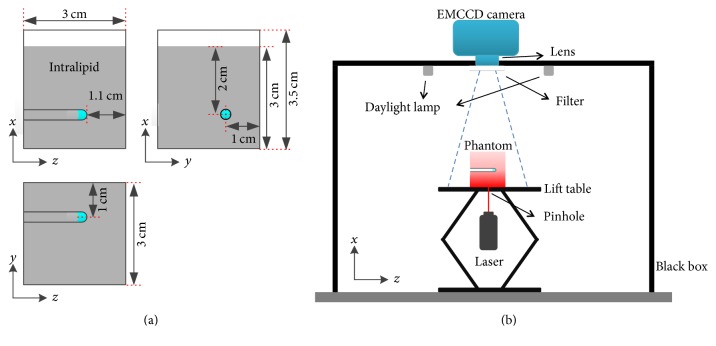
Phantom and imaging system. (a) Geometry of the phantom. (b) Schematic of the transillumination FPI.

Phantom experiments were carried out to validate the proposed image restoration method. The phantom was a 3 × 3 × 3.5 cm^3^ cuboid tank made of perspex as shown in Figure 5(a). The cuboid tank was filled with diluted Intralipid-20% with a volume concentration of 5% and the height of the Intralipid-20% in the tank was 3 cm. A transparent glass capillary tube with a diameter of 0.3 cm was immersed in the tank. Holes were drilled on the wall of the tank with a thickness of 5 mm for the fastening of the tube. The depth of the tube was 2 cm. The distance between the center of the tube and the boundary along *y*-axis was 1 cm. The distance between the bottom of the tube and the boundary along *z*-axis was 1.1 cm. 20 *μ*L Cy5.5 dye with five different concentrations of 4, 6, 8, and 10 *μ*mol/L was successively filled into the tube as the fluorescent target. The fluorescent images of the phantom were acquired with a transillumination FPI system as shown in Figure 5(b). The phantom was placed on a lift table and an electron multiplying charge-coupled device (EMCCD) camera (iXon3 888, Andor Technologies, UK) coupled with a 50 mm f/0.95 lens (DO-5095, Navitar, USA) was placed above the phantom to capture images. A 711 ± 25 nm bandpass filter (BrightLine, Semrock, USA) in front of the camera was used to capture fluorescent image. A 671 nm laser (CrystaLaser LC, Reno, USA) below the lift table was used to excite the fluorescent target within the phantom through a pinhole at the center of the lift table. Two daylight lamps placed on both sides of the camera were used to obtain white light images. The whole imaging system was covered with a black box to block environmental lights.

### 3.2. Mouse Experiment

**Figure 6:**
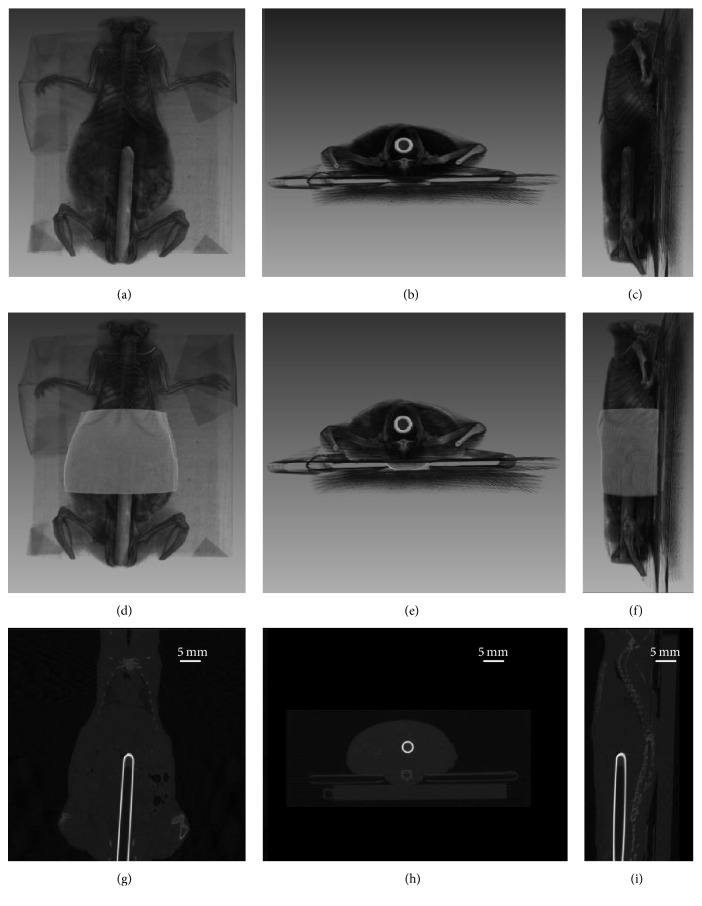
X-CT reconstruction results of the imaged mouse. (a)–(c) Three-dimensional volume rendering of the reconstruction results. (d)–(f) Surface of a section of the mouse torso extracted from X-CT images fused with volume rendering. (g)–(i) Representative slices of the reconstruction results. The first to third columns are the results for coronal, transversal, and sagittal positions, respectively.

For the further validation of the proposed image restoration method, mouse experiment was implemented, which was conducted under the protocol approved by the Fourth Military Medical University Animal Care and Use Committee. The imaged object was a nude mouse. Before the experiment, the mouse was anesthetized with 10% sodium pentobarbital through intraperitoneal injection. A transparent glass capillary tube with a diameter of 0.3 cm filled with 20 *μ*L Cy5.5 dye with a concentration of 1 *μ*mol/L was planted into the abdomen of the mouse. Subsequently, the mouse with a supine position was fastened on a cardboard with black tapes. A slot is located at the center of the cardboard to let excitation light get through. The used FPI system was the same with the phantom experiments. After the acquisition of fluorescent images, X-ray computed tomography (X-CT) scan of the mouse was carried out and the filtered back-projection (FBP) method [48] was applied to accomplish the reconstruction of X-CT. Then the surface of a section of the mouse torso, which is required in the image restoration, was extracted through the segmentations of the X-CT images. The X-CT reconstruction results of the mouse and the extracted surface are shown in Figure 6.

## 4. Results

### 4.1. Phantom Experiments

**Figure 7:**
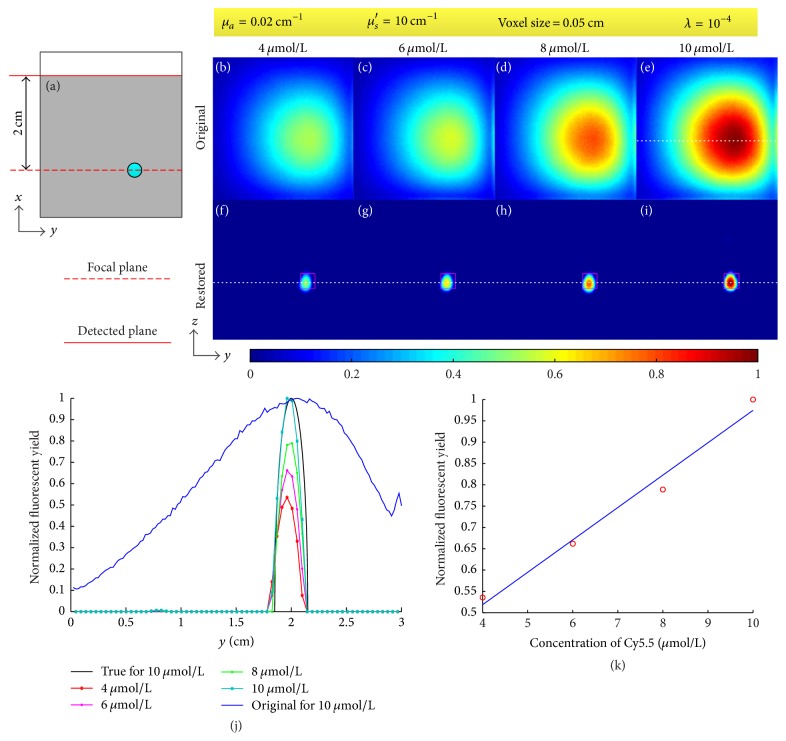
Results of phantom experiments. (a) Illustration of the location of target, focal plane, and detected plane in *xy*-plane. (b)–(e) Original fluorescent images for concentrations of 4, 6, 8, and 10 *μ*mol/L, respectively. (f)–(i) Restored images of (b)–(e) in which the magenta square denotes the true location of target. (j) Profiles along the white dotted line in (e)–(i). (k) Linear fitting of the maximum of (f)–(i). The parameters are given in the top yellow box.

The results of phantom experiments are shown in Figure 7. For all the image restoration results, the depth of focal plane (DFP) was set as 2 cm, the voxel size was set as 0.05 cm, and the optical coefficients *μ*_*a*_ and *μ*_*s*_′ were set as 0.02 cm^−1^ and 10 cm^−1^, respectively. The regularization parameter *λ* was empirically chosen as 10^−4^ and 200 iterations were executed. Figure 7(a) is an illustration of the location of target, focal plane, and detected plane in *xy*-plane. Figures 7(b)–7(e) are the original fluorescent images for concentrations of 4, 6, 8, and 10 *μ*mol/L normalized with the maximum of (e) while Figures 7(f)–7(i) are the corresponding restored images normalized with the maximum of (i). The magenta square in Figures 7(f)–7(i) shows the true location of target. Figure 7(j) provides profiles along the white dotted line in (e)–(i) as well as the true profile. Because the tube is a cylinder, the true profile is a curve with a formula of $g(h)=\sqrt{r^{2}-h^{2}}$, where *r* is the radius of the tube and *h* is the distance away from the center of the tube. Figure 7(k) shows a linear fitting of the maximum of (f)–(i). The full widths at half maximum (FWHMs) of the profiles of the original and the restored images in Figure 7 are shown in Table 1.

**Figure 8:**
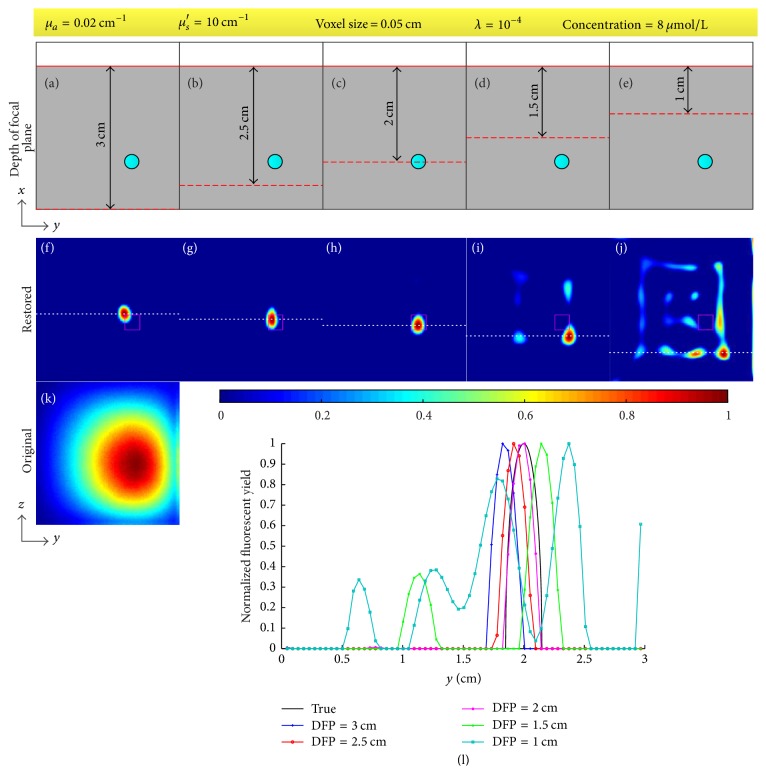
Effect of DFP. (a)–(e) Illustration of the location of target, focal plane, and detected plane in *xy*-plane. (f)–(j) Restored images corresponding to (a)–(e). (k) Original fluorescent image. (l) Profiles along the white dotted line in (f)–(j). The fixed parameters are given in the top yellow box.

**Figure 9:**
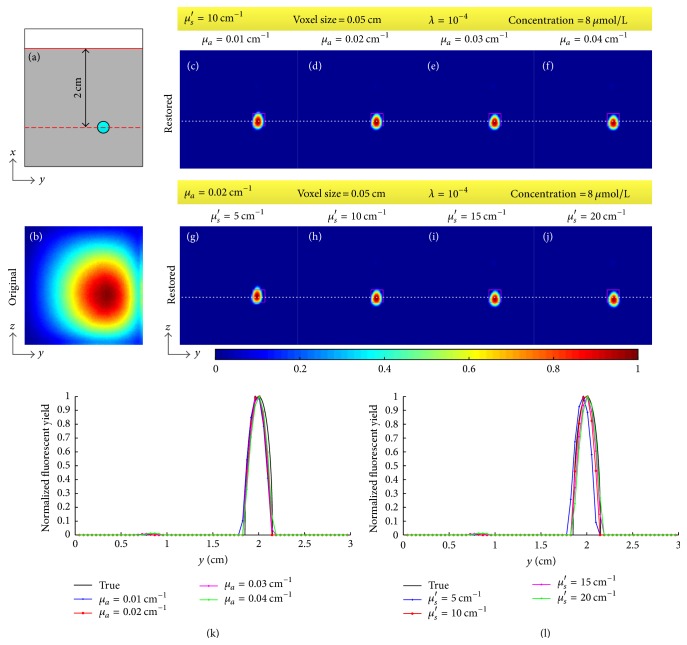
Effect of optical coefficients. (a) Illustration of the location of target, focal plane, and detected plane in *xy*-plane. (b) Original fluorescent image. (c)–(f) Restored images when the absorption coefficients *μ*_*a*_ are 0.01, 0.02, 0.03, and 0.04 cm^−1^, respectively. (g)–(j) Restored images when the reduced scattering coefficients *μ*_*s*_′ are 5, 10, 15, and 20 cm^−1^, respectively. (k) Profiles along the white dotted line in (c)–(f). (l) Profiles along the white dotted line in (g)–(j). The fixed parameters are given in the yellow box.

**Figure 10:**
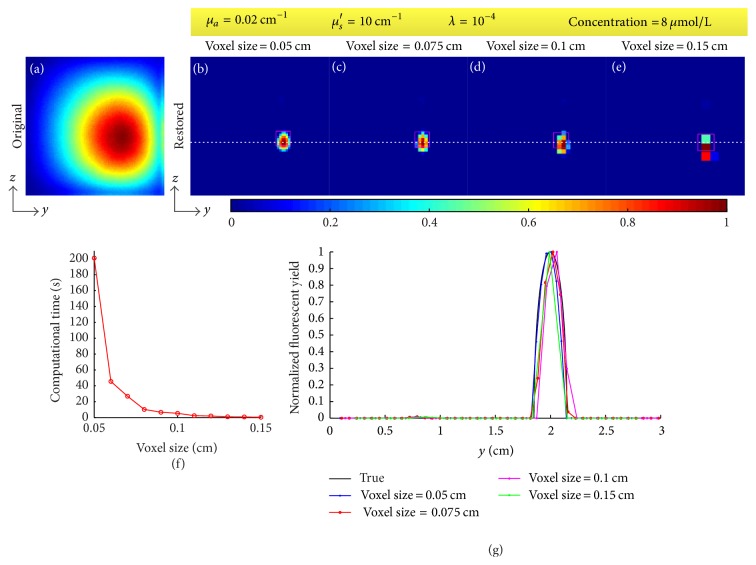
Effect of voxel size. (a) Original fluorescent image. (b)–(e) Restored images when the voxel sizes are 0.05, 0.075, 0.1, and 0.15 cm, respectively. (f) Computational time as a function of voxel size with a sampling interval of 0.01 cm. (g) Profiles along the white dotted line in (c)–(f). The fixed parameters are given in the top yellow box.

**Figure 11:**
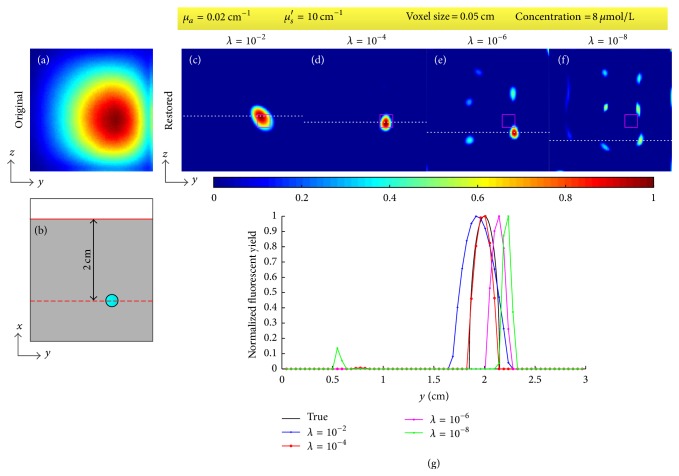
Effect of regularization parameter. (a) Original fluorescent image. (b) Illustration of the location of target, focal plane, and detected plane in *xy*-plane. (c)–(f) Restored images when the regularization parameter *λ* is 10^−2^, 10^−4^, 10^−6^, and 10^−8^, respectively. (g) Profiles along the white dotted line in (c)–(f). The fixed parameters are given in the top yellow box.

The DFP, the optical coefficients, the voxel size, and the regularization parameter affect the results. We tested the effect of the four factors through restoring the fluorescent image for the concentration of 8 *μ*mol/L. During the test of each factor, the tested factor was changed while the other three were set as above (i.e., DFP was 2 cm, *μ*_*a*_ = 0.02 cm^−1^, *μ*_*s*_′ = 10 cm^−1^, voxel size was 0.05 cm, and *λ* = 10^−4^). The two optical coefficients were also tested separately through changing one and fixing another. The results are shown in Figures 8–11.  Figure 8 provides the restored images and profiles when the DFPs are 3 cm, 2.5 cm, 2 cm, 1.5 cm, and 1 cm and Table 2 shows the corresponding deviations of the centers of restored targets from the true center.  Figure 9 gives the restored images and profiles when the absorption coefficients *μ*_*a*_ are set as 0.01, 0.02, 0.03, and 0.04 cm^−1^ and the reduced scattering coefficients *μ*_*s*_′ are set as 5, 10, 15, and 20 cm^−1^.  Table 3 lists the FWHMs of the profiles of restored images with different optical coefficients.  Figure 10 shows the restored images and profiles with voxel sizes of 0.05, 0.075, 0.1, and 0.15 cm and Table 4 provides the corresponding FWHMs as well as the computational time. To show the voxel size in the images, Figures 10(c)–10(f) are shown without interpolation. Computational time as a function of voxel size with a sampling interval of 0.01 cm is also provided as Figure 10(f).  Figure 11 shows the restored images and profiles with regularization parameters of 10^−2^, 10^−4^, 10^−6^, and 10^−8^.  Table 5 lists the corresponding derivations and FWHMs. For Figure 11(f), the lower right bulk is considered as the restored target. All the images are normalized with the maximums.

### 4.2. Mouse Experiment

**Figure 12:**
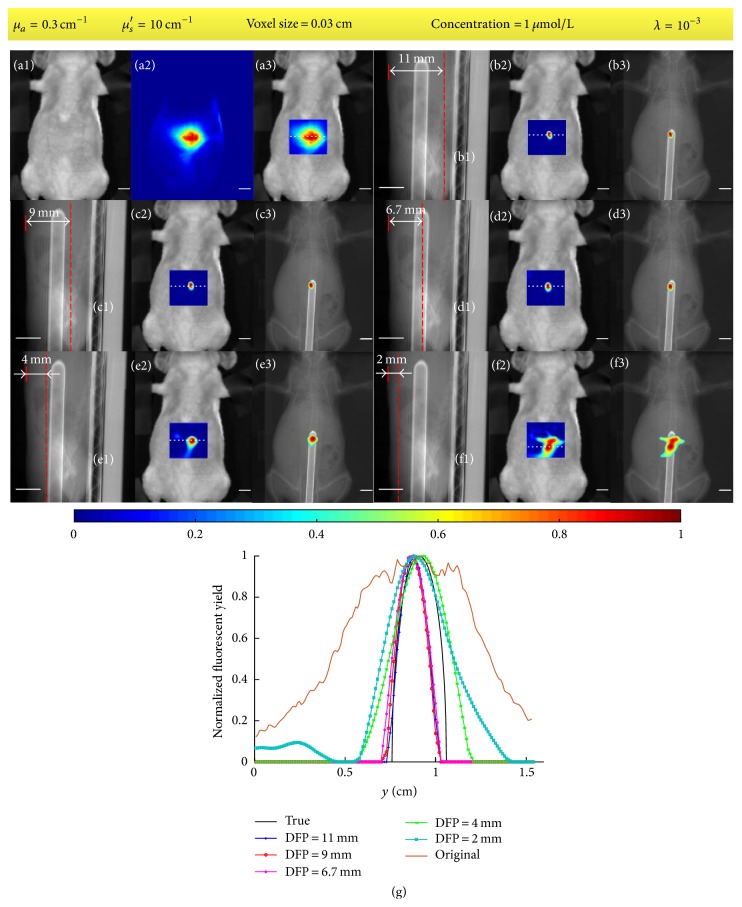
Results of mouse experiment. (a1)–(a3) White light image, original fluorescent image, and a fused image of them, respectively. (b1), (c1), (d1), (e1), and (f1) Illustrations of the locations of focal planes on a part of a sagittal X-CT projection image. (b2), (c2), (d2), (e2), and (f2) Restored images fused with white light images when the DFPs are set as 1.1 cm, 0.9 cm, 0.67 cm, 0.4 cm, and 0.2 cm, respectively. (b3), (c3), (d3), (e3), and (f3) Corresponding restored images fused with a coronal X-CT projection image. (g) Profiles along the white dotted line in (a3), (b2), (c2), (d2), (e2), and (f2). All the scale bars denote 0.5 cm. The fixed parameters are shown in the yellow box.

The results of mouse experiment are shown in Figure 12. A part of the torso of the mouse with a size of about 1.8 × 2.9 × 2 cm^3^ was used to model the light propagation. For fine images, a smaller voxel size, 0.03 cm, than the values in the phantom experiments was chosen. The optical coefficients *μ*_*a*_ and *μ*_*s*_′ were set as 0.3 cm^−1^ and 10 cm^−1^, respectively. The regularization parameter was empirically chosen as 10^−3^ and the image restorations were terminated after 200 iterations. The white light image, original fluorescent image, and a fused image of them are given in Figures 12(a1)–12(a3). Five different DFPs (1.1 cm, 0.9 cm, 0.67 cm, 0.4 cm, and 0.2 cm) were tested and the corresponding results are shown as Figures 12(b1)–12(f3). The results are shown with the restored image fused with the white light image as well as a coronal X-CT projection image. Profiles along the white dotted line in Figures 12(a3), (b2), (c2), (d2), (e2), and (f2) are shown in Figure 12(g). All the images are normalized with the maximums.

## 5. Discussion

**Figure 13:**
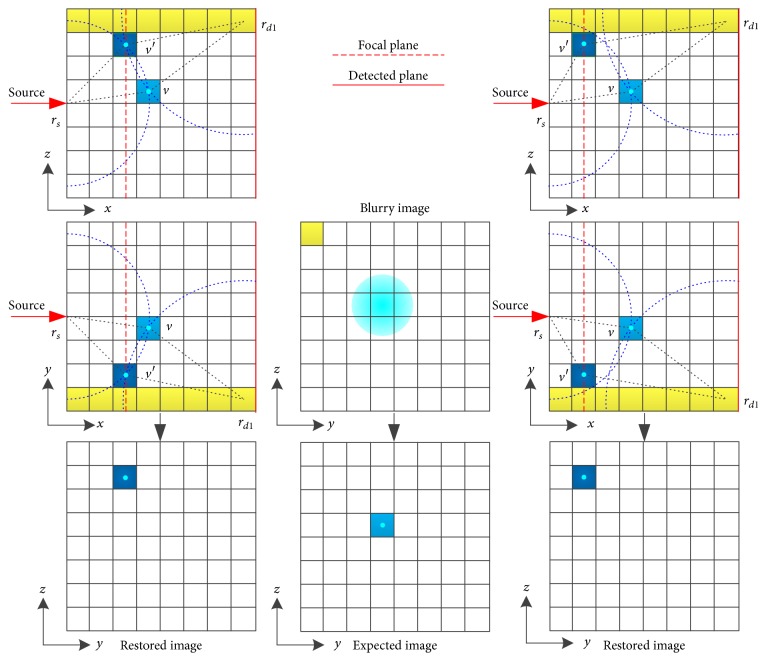
Illustration of the effect of DFP with an 8 × 8 image and a point target. The left column shows the case when the intersection of the focal plane and the circles with *r*_*s*_ and *r*_*d*1_ as their centers exists. The right column shows the case when the intersection does not exist. The middle column shows the blurry image and the expected image. The pixel in light blue denotes the true location of target while the pixel in dark blue denotes the location of virtual target in the focal plane.

**Figure 14:**
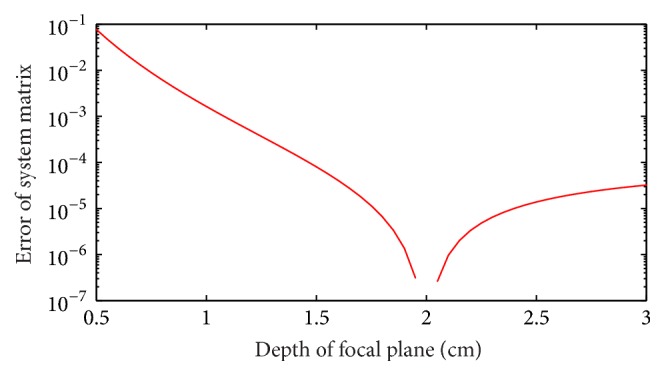
Error of system matrix as a function of DFP for the phantom experiments. The depth of target is 2 cm.

Figure 7 and Table 1 show that the proposed method is capable of restoring the blurry images caused by photon diffusion when the DFP equals the depth of target. The profiles in Figure 7(j) and Table 1 demonstrate that the size of the restored fluorescent target in terms of FWHM (~0.22 cm) is close to the size of the true target (0.3 cm) while the FWHMs of the original fluorescent images are around 1.86 cm. Figure 7(k) indicates the pixel values of the restored images are approximately proportional to the concentration of Cy5.5 dye. The quality of image restoration relies on the choice of DFP as shown in Figure 8 and Table 2. When the DFP is deeper than the depth of target, the target can also be well restored but the restored location deviates from the true location as shown in Figures 8(f) and 8(g) as well as the deviations listed in Table 2. The deeper the DFP is, the farther the restored location deviates from the true location. For example, the deviation for a DFP of 3 cm is 0.26 cm but 0.10 cm for a DFP of 2.5 cm. On the other hand, when the DFP is shallower than the depth of target, the restored target tends to be spread around the image as shown in Figures 8(i) and 8(j). As referred in the derivation of (10), the fluorescent signals are conceived to come from the focal plane. Consequently, if the focal plane is not located at the true depth of target, the image restoration will result in a virtual target in the focal plane and the location of this virtual target varies with DFP. From (10) and (6), we know that the restored image *F*^(1)^ corresponds to the first pixel of the blurry image and the weight of each voxel *C*_*n*,*i*_^(*m*)^ = *G*_em_(*r*_*dm*_, *x*_*i*_, *y*_*n*_, *z*_*n*_)Φ_ex_(*x*_*i*_, *y*_*n*_, *z*_*n*_, *r*_*s*_) is a function of the location of the pixel of the blurry image *r*_*dm*_, the location of source *r*_*s*_, and the location of voxel (*x*_*i*_, *y*_*n*_, *z*_*n*_). If we ignore the effect of boundary condition, Φ_ex_ and *G*_em_ are functions of the distance between *r*_*s*_ and (*x*_*i*_, *y*_*n*_, *z*_*n*_) and the distance between *r*_*dm*_ and (*x*_*i*_, *y*_*n*_, *z*_*n*_) [45]. We use Figure 13 to illustrate the effect of DFP, where the fluorescent target is assumed to be a point. In Figure 13, the problem is illustrated on *xz*-plane and *xy*-plane separately. *v* and *v*′ denote the locations of the true target and the virtual target, respectively. Because the restored image *F*^(1)^ corresponds to the first pixel of the blurry image, we only consider the situation *r*_*dm*_ = *r*_*d*1_. In order to achieve the same pixel value *B*_1_, the weights of the true target and the virtual target should be the same; that is, the distance $\bar{r_{s}v}$ should equal $\bar{r_{s}v^{\prime }}$ and $\bar{r_{d1}v}$ should equal $\bar{r_{d1}v^{\prime }}$. Therefore, the virtual target should be located at the intersection of the focal plane and the circles with *r*_*s*_ and *r*_*d*1_ as their centers as shown in the left column in Figure 13. However, the intersection does not exist for most situations as shown in the right column in Figure 13. In these situations, the voxel within the circle intersecting the focal plane and with a weight closest to the weight at *v* could be considered as the virtual target. The above analysis ignores the effect of boundary condition and the target is a point. The actual situation is much more complicated due to the boundary of the imaged object as well as the volume of the fluorescent target. We analyze the location of the virtual target through finding an equivalent of the true target within the focal plane. However, the virtual target may not be well restored because the weight of the virtual target may be quite different from the one of the true target.  Figure 14 provides the error of system matrix (‖*R* − *R*_true_‖_2_^2^) as a function of DFP for the phantom experiments, where the depth of target is 2 cm and the system matrix with a DFP of 2 cm is assumed as the true system matrix. It can be observed in Figure 14 that the error of system matrix with a shallower focal plane rises much faster than the one with a deeper focal plane. This explains why the target cannot be well restored when the DFP is shallower than the depth of target in Figures 8(i) and 8(j). The results of mouse experiment in Figure 12 and Table 6 are consistent with the results of phantom experiments in Figure 8. Because of the tiny size of the mouse, the locations of the restored target vary slightly when the DFP is deeper than the true depth of target in Figure 12. In general, Figures 7, 8, and 12 as well as Tables 1, 2, and 6 indicate the proposed method is capable of restoring the blurry images caused by photon diffusion when the DFP is set properly. The target can be well revealed when the DFP is within an interval around the true depth of target rather than an exact value.

Generally, the proposed method is more appropriate for the cases in which the depths of targets can be estimated because the location of the restored target varies with DFP. This restricts the applications of the proposed method. On the contrary, we might take use of the effect of DFP to estimate the depth of target. It is known that commonly the location of the maximum of the blurry image should correspond to the center of the fluorescent target. Therefore, we could restore the blurry image with a set of DFPs ranging from 0 to the thickness of the imaged object and compare the center of the restored images with the center of the blurry image to estimate the depth of the target.

The optical coefficients *μ*_*a*_ and *μ*_*s*_′ are prerequisites for the image restoration. In the phantom experiments, we used the diluted Intralipid-20% with a volume concentration of 5% as the diffusion medium, the absorption coefficient and reduced scattering coefficient of which are, respectively, around 0.02 cm^−1^ and 10 cm^−1^ when the wavelength is between 632.8 nm and 751 nm [49]. Therefore, we consider 0.02 cm^−1^ and 10 cm^−1^ as the true optical coefficients of the diffusion medium and tested the effect of the optical coefficients through setting them as 50%, 100%, 150%, and 200% of the true values. The results in Figure 9 and Table 3 indicate that the effect of optical coefficients is slight (FWHMs are around 0.22). Based on this conclusion, we used homogeneous optical coefficients in the mouse experiment rather than a heterogeneous model.

The results of the image restoration greatly depend on the voxel size as shown in Figure 10 and Table 4. In general, a small voxel size would result in a fine image but consumes more time on computation and more memories on the storage of system matrix and depth conversion matrices. For example, when the imaged object is a 3 × 3 × 3 cm cube and the voxel size is 0.05 cm, the image size will be 61 × 61 and the number of voxels will be 61 × 61 × 61. It results in the fact that the size of system matrix is 3,721 × 3,721 and the size of each depth conversion matrix is 3,721 × 61. When the data are saved with double precision, the system matrix and each depth conversion matrix will occupy about 105.6 and 1.7 megabytes of memories, respectively. For the storage of all the depth conversion matrices and the system matrix, about 6.4 gigabytes is required. To reduce the requirement of the memories, we can save only the elements of depth conversion matrices required during the calculation of system matrix. Moreover, the calculations of system matrix focus on the calculations of weights *C*_*n*,*i*_^(*m*)^, that is, the calculations of *G*_em_ and Φ_ex_. For the above case, it is required to calculate *G*_em_ and Φ_ex_ 61 × 61 × 61 + 61 × 61 × (61 × 61 − 1) times, that is, 14,069,101 times at least for a single DFP. In parallel, if the voxel size is 0.1 cm, the sizes of system matrix and each depth conversion matrix will be 961 × 961 and 961 × 31, respectively, and *G*_em_ and Φ_ex_ will be calculated 952,351 times for a single DFP. Through Figure 10(f) and Table 4, it can be found that the computational time exponentially increases with the decrease of the voxel size. For example, the computational time is only 0.5 seconds for a voxel size of 0.15 cm but 201 seconds for a voxel size of 0.05 cm; that is to say, when the voxel size reduces 3-fold, the computation time increases about 400-fold. This limits the choice of voxel size. In general, the voxel size should be as small as possible when the computational time and memories are not limited.

Figure 11 and Table 5 show the effect of regularization parameter. The function of the regularization parameter is controlling the effect of regularization. The regularization aims to suppress the impact of ill-posedness and noise through smoothing the solution vector. A large regularization parameter will result in oversmoothness like Figure 11(c) with a FWHM of 0.39 cm; on the contrary, a small one may lead to image distortion because of noise like Figures 11(e) and 11(f) with deviations of 0.30 cm and 0.51 cm. The choice of the regularization parameter depends on the noise level of data. Due to the fact that noise level is unable to be achieved in some applications, the regularization parameter is usually determined empirically [50, 51].

A defect of the proposed method is the requirement of the geometry of the imaged object, which is not necessary in FPI. The geometry is a prerequisite for modeling the photon propagation in the imaged object. Thus, an additional imaging modality such as X-CT and magnetic resonance imaging (MRI) is indispensable. As an alternative, the imaged object can be immersed into a tank with regular geometry filled with matching fluids.

Although the proposed method in this paper is based on a transillumination FPI system, it may be feasible for epi-illumination mode in theory as long as the location of source is available. If the source is still a point source, the methods for the two modes have no difference except the locations of source. When a planar source is implemented, the distribution of the photon density for excitation Φ_ex_ is not a Green's function solution to the diffusion equation anymore but a convolution of Green's function and the distribution function of the source. In general, transillumination mode is more appropriate because the aim of the proposed method is the imaging of deep fluorescent targets while transillumination mode can easily excite deep fluorochromes.

The proposed method is conceived based on the reconstruction scheme in FMT. It is well known that FMT reconstructs a three-dimensional distribution of fluorescent yield with multiple projection images from different angles, while the proposed method can be considered as the reconstruction of a two-dimensional image from a single blurry image. It is infeasible for the reconstruction method in FMT to directly reconstruct the distribution of fluorescent yield from a single image because the mismatch between the quantities of data and unknown parameters will result in great degradation of reconstruction results [52]. Through the definition of a new unknown parameter *F*_*n*_ in (6), the proposed method balances the quantities of data and unknown parameters to make the reconstruction possible. In addition, although FMT can provide three-dimensional distribution of fluorescent yield, it cannot displace FPI because of the stability, simplicity, convenience, and fast data acquisition of FPI. Therefore, there are still quantities of applications accomplished with FPI especially those requiring high data acquisition speed such as pharmacokinetics [16, 17].

The results of phantom and mouse experiments prove that the proposed image restoration method is capable of revealing fluorescent targets beneath diffusion medium. It is well known that the near-infrared light is highly scattered within the tissues of living bodies and it is difficult to capture the shapes of deep fluorescent targets beneath tissues with cameras. This limits the applications of FPI. For example, size of tumor is an important indicator in oncology. Fluorescence planar imaging is a usual imaging technique for the research of tumor on animal model through fluorescent molecular probes. However, the estimation of size of tumor through FPI images is feasible for only subcutaneous tumor while the size of in situ tumor is difficult to be estimated. The proposed method provides the ability of the estimation of size of deep fluorescent targets like in situ tumors.

## 6. Conclusion

In conclusion, an image restoration method is proposed to deal with the blurring caused by photon diffusion in FPI. The method is conceived based on the reconstruction scheme in FMT. The primary contribution of this work is the construction of system matrix, which is achieved through the definition of a new unknown parameter and depth conversion matrices with a chosen focal plane. The new unknown parameter is defined through the first mean value theorem for definite integrals and represents a weighted average of the fluorescent yields along the detection direction. Results of phantom and mouse experiments indicate that the proposed image restoration method is capable of reducing the blurring of fluorescent image caused by photon diffusion when the depth of focal plane is chosen within a proper range around the true depth of fluorochrome. The effect of optical coefficients is slight. The quality of the restored image greatly depends on the voxel size but it is limited by the computational time and memories. The regularization parameter also influences the results that a large regularization parameter would result in oversmoothness while a small one might lead to image distortion. This method will be helpful to the estimation of the size of deep fluorochrome.

## Figures and Tables

**Table 1:** Full widths at half maximum of the profiles of the original and restored images with different concentrations of Cy5.5.

Concentration (*μ*mol/L)	4	6	8	10
FWHM (cm)				
Restored	0.21	0.21	0.22	0.22
Original	1.86	1.85	1.86	1.87

**Table 2:** Deviations of the centers of restored targets from the true center with different DFPs.

DFP (cm)	3	2.5	2	1.5	1
Deviation (cm)	0.26	0.10	0.04	0.31	0.74

**Table 3:** Full widths at half maximum of the profiles of restored images with different optical coefficients.

*μ*_*a*_ (cm^−1^)	0.01	0.02	0.03	0.04	0.02	0.02	0.2	0.02
*μ*_*s*_′ (cm^−1^)	10	10	10	10	5	10	15	20
FWHM (cm)	0.21	0.22	0.22	0.21	0.22	0.22	0.22	0.21

**Table 4:** Full widths at half maximum of the profiles of restored images and computational time with different voxel sizes.

Voxel size (cm)	0.05	0.075	0.1	0.15
FWHM (cm)	0.22	0.20	0.19	0.15
Computational time (s)	201.0	23.1	5.4	0.5

**Table 5:** Deviations of the centers of restored targets from the true center and FWHMs of the profiles of restored images with different regularization parameters.

*λ*	10^−2^	10^−4^	10^−6^	10^−8^
Deviation (cm)	0.13	0.04	0.30	0.51
FWHM (cm)	0.39	0.22	0.16	0.10

**Table 6:** Full widths at half maximum of the profiles of restored images in mouse experiment with different DPFs.

DFP (cm)	0.2	0.4	0.67	0.9	1.1	Original
FWHM (cm)	0.40	0.35	0.22	0.19	0.18	0.88
